# Diagnostic Utility of the Ocular Surface Test for Psoriasis-Associated Conjunctivitis: A Case Report

**DOI:** 10.7759/cureus.88619

**Published:** 2025-07-23

**Authors:** Rumi Adachi, Jun Shoji, Akiko Tomioka, Noriko Inada, Satoru Yamagami

**Affiliations:** 1 Division of Ophthalmology, Department of Visual Sciences, Nihon University School of Medicine, Tokyo, JPN

**Keywords:** biomarker, conjunctivitis, il-22, ocular surface test, psoriasis

## Abstract

Psoriasis-associated conjunctivitis, a rare eye complication in patients with psoriasis, is not always accurately diagnosed. The symptoms may become prolonged, making treatment challenging. We report the case of a patient with psoriasis-associated conjunctivitis for whom we identified biomarkers for psoriasis in an ocular surface test. A 64-year-old female patient presented with refractory conjunctivitis that recurred despite treatment with a combination of steroids and antimicrobial eye drops and dry eye treatment. She had a long history of symptoms of psoriasis without treatment. Slit-lamp examination at the initial visit to our hospital revealed severe conjunctival hyperemia without conjunctival papillae or follicles and punctate superficial keratitis in the inferior area of the cornea. We initiated treatment with betamethasone ophthalmic solution and tacrolimus ointment for eyelid application, and her conjunctivitis improved one week following the initial visit. An ocular surface test was performed to measure the levels of cytokine and chemokine mRNA expression and profile of inflammasome-associated factors on the ocular surface. When comparing the specimens obtained at the initial visit and those taken one week later, interleukin (IL)-17A and IL-22 were expressed in the right eye, and IL-1β, prostaglandin-endoperoxide synthase 2 (PTGS2/COX-2), NOD-like receptor family CARD domain-containing protein 4 (NLRC4), Familial Mediterranean Fever (MEFV) gene, and caspase 5 (CASP5) decreased by more than six-fold. Changes in biomarkers associated with psoriasis pathology can be observed in ocular surface tests of patients with psoriasis-associated conjunctivitis.

## Introduction

Psoriasis is a chronic inflammatory skin disease accompanied by lichenification, with a pathogenesis that is thought to involve autoimmunity. The incidence of psoriasis is 1-2% worldwide. Ocular complications develop in 10-12% of patients with psoriasis [1]. The levels of interleukin (IL)-17 and IL-23 increase in skin lesions of patients with psoriasis, and the disease is mainly a Th17-type inflammatory disease [2]. Increased IL-22 expression in psoriatic skin lesions correlates with the psoriasis area and severity index (PASI), a measure of psoriatic skin severity, suggesting that psoriatic skin lesions depend on IL-22 expression in addition to the IL-23/Th17 axis [3]. Uveitis is a well-known ocular complication. Patients with psoriasis can develop conjunctivitis; however, clinical findings of conjunctivitis are nonspecific, requiring differential diagnosis from infectious and noninfectious conjunctivitis other than psoriasis-associated conjunctivitis. The clinical examinations for accurately diagnosing psoriasis-associated conjunctivitis are not well-established in ophthalmology, and its ocular pathology remains unknown. Herein, we report the case of a patient with suspected psoriatic conjunctivitis who exhibited increased expression of psoriasis-related cytokines and inflammasome mRNA in an ocular surface test.

## Case presentation

A 64-year-old female patient presented to our clinic with complaints of repeated redness and eye discharge. Her medical history included psoriasis, and her symptoms appeared in her 20s. Psoriasis treatment was interrupted due to frequent job transfers, and she was unable to continue receiving appropriate treatment. One year prior, she developed severe hyperemia and eye discharge similar to epidemic keratoconjunctivitis. A local ophthalmologist had treated her for acute conjunctivitis and dry eyes. Although the symptoms disappeared following treatment with diquafosol sodium ophthalmic solution, sodium hyaluronate ophthalmic solution 0.1%, gatifloxacin ophthalmic solution, and fluorometholone ophthalmic solution 0.1%, they recurred repeatedly at 1-2 week intervals. The patient was referred to our department for evaluation and treatment.

**Figure 1:**
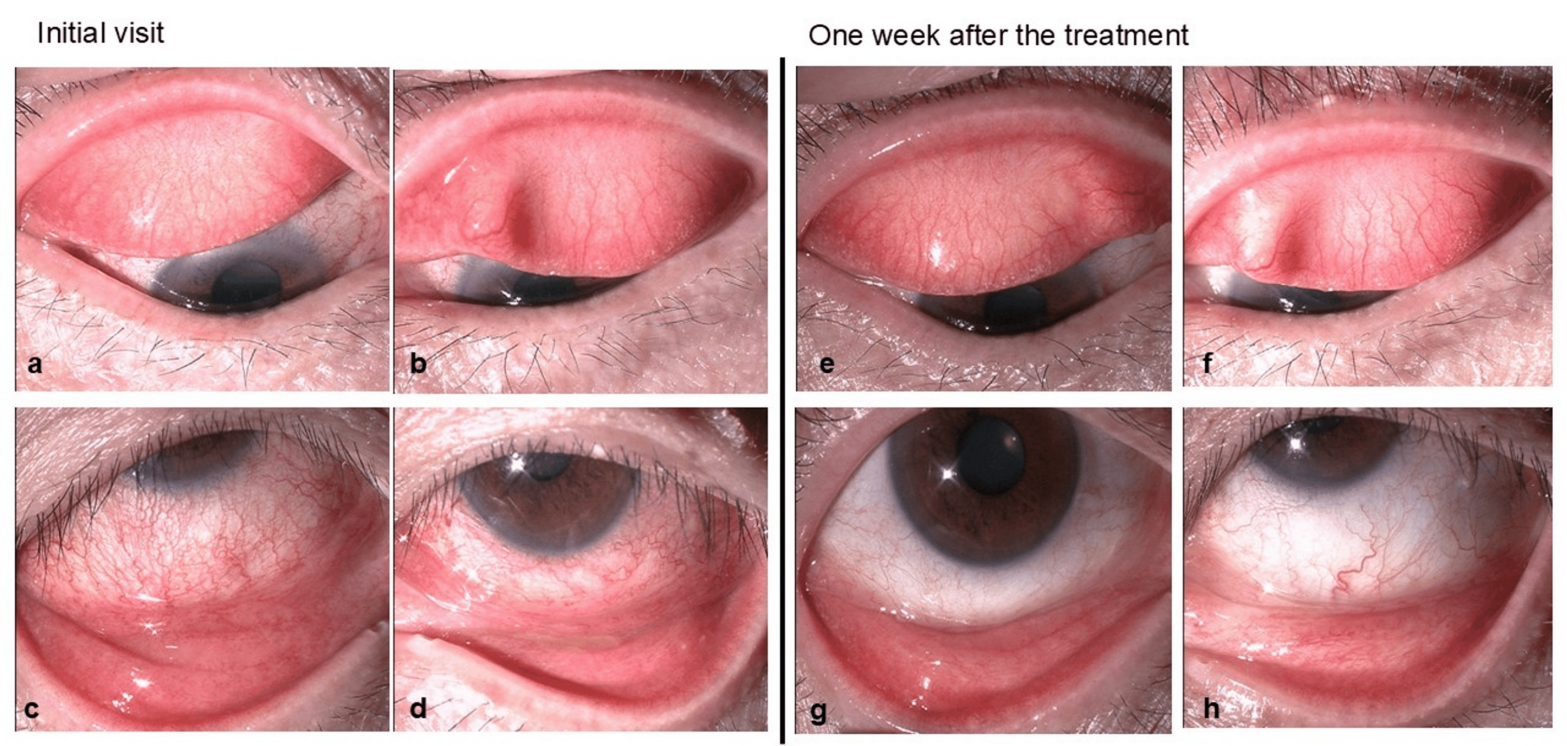
Anterior segment of slit-lamp photographs at the initial visit and one week after the treatment. Initial visit (a-d). (a) Right upper palpebral conjunctiva. (b) Left upper palpebral conjunctiva. (c) Right lower palpebral conjunctiva and bulbar conjunctiva. (d) Left lower palpebral conjunctiva and bulbar conjunctiva. The palpebral conjunctivae of both eyes showed moderate hyperemia (grade 3) and swelling. No conjunctival follicles or papillae were observed. The bulbar conjunctivae of both eyes showed moderate hyperemia (grade 3). One week post-treatment (e-h). (e) Right upper palpebral conjunctiva. (f) Left upper palpebral conjunctiva. (g) Right lower palpebral conjunctiva and bulbar conjunctiva. (h) Left lower palpebral conjunctiva and bulbar conjunctiva. Hyperemia (grade 1) and swelling in the palpebral conjunctivae of both eyes were resolved. Hyperemia (grade 1) in both bulbar conjunctivae also improved.

At the initial visit, the conjunctiva was hyperemic (grade 3 by slit-lamp findings classification scale [4]); however, there were no conjunctival papillae or follicles (Figures 1a-1b). The bulbar conjunctiva was hyperemic (Figures 1c-1d). Conjunctival hyperemia was more severe in the right than the left eye. Punctate superficial keratitis was observed in the inferior cornea. No inflammatory or uveitis findings were observed in the anterior chamber. The differential diagnosis included infectious and allergic conjunctivitis, but based on the patient’s background of untreated psoriasis, we clinically diagnosed psoriasis-associated conjunctivitis. The following clinical examinations were performed for differential diagnosis. The patient was diagnosed with psoriasis vulgaris after consultation with a dermatologist at our hospital. Clinically, her psoriasis lesions had been present for 40 years, with scales and erythema on her abdomen and keratotic erythema on her back. Bacterial culture tests of conjunctival smears detected *Serratia marcescens* in the right eye and *Cutibacterium acnes* and coagulase-negative *Staphylococcus* in the left eye; however, each amount of bacteria detected was extremely small, and these were not determined to be the causative bacterium of conjunctivitis. In the specific allergen test conducted using the multi-antigen simultaneous test (MAST-36; Hitachi Chemical, Tokyo, Japan), the allergens that tested positive for antigen-specific IgE antibodies in serum were house dust mites, house dust, and Japanese cedar. Treatment with betamethasone ophthalmic solution three times a day was initiated, with tacrolimus ointment for eyelid application once a day. At the one-week follow-up examination, conjunctival hyperemia and bulbar conjunctival hyperemia (grade 1) had improved (Figures 1e-1h) and betamethasone ophthalmic eye drops were completed; application of tacrolimus ointment once a day to the eyelids was continued. During the month that we continued treatment, there were no recurrences of conjunctivitis. Since then, the patient has been undergoing treatment with tacrolimus ointment while visiting a local doctor for two months, and we have not received any reports of recurrence.

Ocular surface test

We performed an ocular surface test to analyze the ocular surface pathology and confirm the diagnosis. Our ocular surface test quantitatively measured the mRNA expression of markers expressed on the ocular surface by collecting ocular surface-coated tears, mucus, and conjunctival epithelium using a filter paper-based impression cytology method [5]. Specimens were obtained from the upper palpebral conjunctiva using impression cytology at the initial visit and again one week later. The mRNA was extracted from the specimen, and levels of cytokines and chemokines, inflammasome profiles, and goblet cell markers on the ocular surface were analyzed.

First, we used quantitative reverse transcription polymerase chain reaction (RT-PCR) to measure the mRNA levels of IL-4, Th2-type inflammation-related cytokines, IL-17A and IL-22, Th17-type inflammation-related cytokines, and SAM-pointed domain-containing ETS-like factor (SPDEF), a differentiation marker of goblet cells, using the TaqMan gene expression assay (Life Technologies Japan, Tokyo, Japan), and compared the levels before and after treatment.

**Figure 2:**
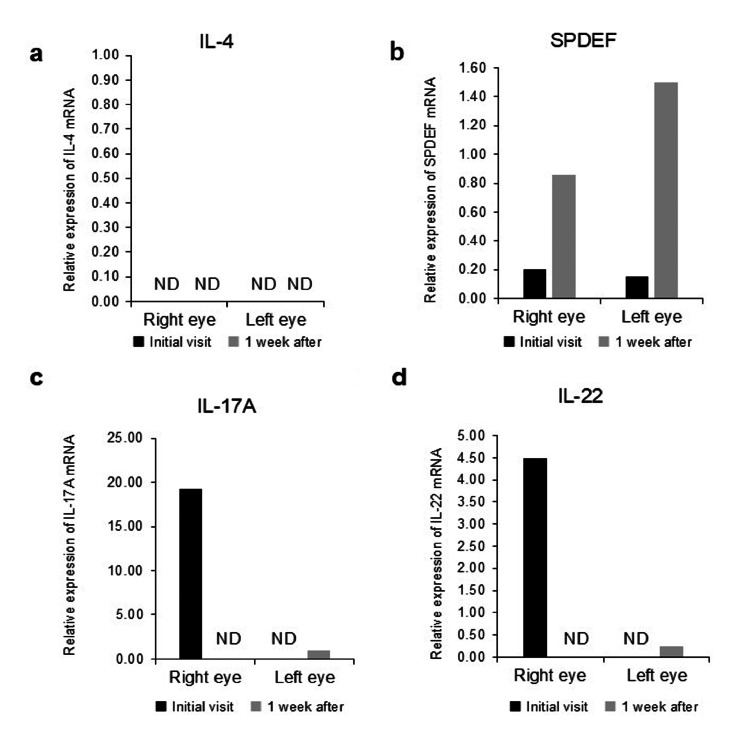
Comparison of mRNA expression levels on the ocular surface at the initial visit and one week later. (a) IL-4, (b) SPDEF, (c) IL-17A, and (d) IL-22. The mRNA expression of IL-4, IL-17A, and IL-22 decreased one week post-treatment compared to the initial visit. IL: interleukin; ND: not detected; SPDEF: SAM-pointed domain-containing ETS-like factor

The results of the ocular surface tests for cytokines and chemokines are shown in Figures 2a-2d. In the specimens obtained before treatment, IL-17A and IL-22 mRNA were highly expressed in the right eye but not in the left. IL-4 was not expressed before or after treatment. SPDEF mRNA levels in specimens before treatment were higher than in specimens after.

Second, we used mRNA extracted from impression cytology specimens to examine the inflammasome profile using the polymerase chain reaction (PCR) array method (RT² Profiler PCR Array Human Inflammasomes; Qiagen, Frederick, USA). The PCR array method enables the examination of five housekeeping genes and 84 inflammasome-related genes. The factors with more than six-fold difference before and after treatment in the PCR array results are presented in Table 1. These factors were IL-1β, prostaglandin-endoperoxide synthase 2 (PTGS2/COX-2), NOD-like receptor family CARD domain-containing protein 4 (NLRC4), Familial Mediterranean Fever (MEFV) gene, and caspase 5 (CASP5). 

**Table 1:** Results of PCR array. IL: interleukin; PTGS2/COX-2: prostaglandin-endoperoxide synthase 2; NLRC4: NOD-like receptor family CARD domain-containing protein 4; MEFV, Familial Mediterranean Fever; CFLAR: caspase 8 and FADD-like apoptosis regulator; CASP5: caspase 5; PCR: polymerase chain reaction

Gene	Fold change (before vs after the treatment)
		IL-1β	85.53
PTGS2/COX-2	32.95
NLRC4	10.26
MEFV	8.04
CFLAR	6.61
CASP5	6.14

## Discussion

This report presents the case of a female patient with severe relapsing conjunctivitis diagnosed with psoriasis-associated conjunctivitis based on the clinical findings and ocular surface test results.

Constantin et al. reported that ophthalmologic changes associated with psoriasis include dry eye disease (18.75% patients with psoriasis), blepharitis, conjunctivitis, cataracts, and uveitis (7-25%) [1]. In this case, punctate superficial keratitis was clinically observed, and the patient had a history of treatment for dry eyes. We measured the goblet cell marker SPDEF to evaluate dry eye and found an increase in SPDEF mRNA expression levels at one week post-treatment. We have reported that the expression level of SPDEF mRNA, a differentiation marker of goblet cells, correlates with MUC5AC mRNA in ocular surface tests of patients with atopic dermatitis [6]. Therefore, the reduced expression of SPDEF mRNA in the corneal surface indicates a decrease in secretory mucins, including MUC5AC. These results suggest that psoriasis-associated conjunctivitis is accompanied by a mucin deficiency dry eye, and that dry eye may improve with treatment for conjunctivitis and blepharitis. However, in this case, the patient's dry eyes, including meibomian gland dysfunction, were not adequately evaluated. Therefore, further investigation into the pathology of dry eyes remains warranted.

Regarding Th17/Th22 responses, IL-17A and IL-22 mRNA expression levels increased in the right eye, which had more severe conjunctivitis, than in the left. The mRNA of IL-22 is expressed at higher levels in skin lesions of patients with psoriasis than in healthy individuals [7]. Similar to psoriasis, Th22-type inflammation influences the severity of atopic dermatitis [8]. In ophthalmology, we have previously reported that IL-22 levels increase in dupilumab-associated conjunctivitis (DAC) and that the levels of IL-22 mRNA expression are useful for diagnosing DAC in the ocular surface test [9]. In IL-22-associated diseases, including psoriasis and atopic dermatitis, IL-22 may induce conjunctivitis. In addition, the mRNA expression of IL-17A was increased in the right eye with severe conjunctivitis. IL-17A is involved in dermatitis, arthritis, gastrointestinal inflammation, the central nervous system, and cardiovascular inflammation in psoriasis [10]; however, data on its involvement in conjunctival diseases remain limited. Both IL-22 and IL-17A may be involved in the pathophysiology, but the clinical findings and pathology of IL-22 and IL-17A-associated conjunctivitis have not been fully elucidated and require further research. The mRNA of IL-4, a Th2-type inflammation-related cytokine, was not expressed before or after treatment. These results also suggest that the conjunctivitis in our cases may be Th17/Th22-associated rather than Th2-associated conjunctivitis.

The inflammasome profile of the ocular surface of our patient with psoriasis-associated conjunctivitis was investigated using the PCR array method. The results showed more than six-fold change in IL-1β, PTGS2/COX-2, NLRC4, MEFV, and CASP5 before and after treatment. IL-1β [11], NLRC4 [12], MEFV [13], and CASP [5,11] have been reported as inflammasomes associated with psoriasis. The results of this study suggest that an inflammatory reaction similar to that of skin lesions occurs in the conjunctiva of patients with psoriasis. Since this is a single-case report, further studies involving a larger number of patients are necessary to confirm the usefulness of this biomarker in diagnosing psoriatic conjunctivitis.

This case report has some limitations. First, reported ocular surface test results included a small sample size (n=1), lacked control comparisons, and had potential variability in impression cytology techniques. Second, fluorescein staining for ocular surface revealed superficial punctate keratitis, suggesting the possible presence of concomitant dry eye. However, detailed tests related to dry eye, including the Schirmer test and tear break-up time (BUT), were not performed, so the presence or absence of concomitant dry eye remains unclear.

## Conclusions

In conclusion, in patients with psoriasis-associated conjunctivitis, an ocular surface test can reveal an inflammatory response similar to that observed in psoriatic skin lesions. Therefore, the factors, including IL-17A, IL-22, IL-1β, NLRC4, MEFV, and CASP5, detected by ocular surface test in our case should be verified for their usefulness as biomarkers for psoriasis-associated conjunctivitis in clinical studies involving a large number of cases.
